# Prevalence and Determinants of Complete Postnatal Care Service Utilization in Northern Shoa, Ethiopia

**DOI:** 10.1155/2018/8625437

**Published:** 2018-08-14

**Authors:** Mohammed Akibu, Wintana Tsegaye, Tewodros Megersa, Sodere Nurgi

**Affiliations:** ^1^Department of Midwifery, Institute of Medicine and Health Sciences, Debre Berhan University, Debre Berhan, Ethiopia; ^2^Concordia University, Montreal, Quebec, Canada; ^3^Obstetrics Unit, Gandhi Maternal Specialized Hospital, Addis Ababa, Ethiopia

## Abstract

**Background:**

Postnatal period presents the highest risk of death for mothers and newborns. Although progress has been made in expanding the coverage for most of maternal health services, national prevalence of postnatal care service utilization in Ethiopia is still extremely limited. Hence, this study aims to determine the prevalence and factors associated with complete postnatal care service utilization in Northern Shoa, Ethiopia.

**Methods:**

Community based cross-sectional survey was conducted between November 2016 and February 2017. A total of 510 mothers were included in the study using multistage sampling technique. The data were collected through face-to-face interview. Bivariate and multivariate logistic regression models were fitted to identify factors associated with complete postnatal care utilization at p value of < 0.05. SPSS version 20 was used to analyze the data.

**Results:**

The prevalence of complete postnatal care utilization was found to be 28.4% in the study area. Mode of delivery (AOR=5.7, 95% CI = 3.9, 19), number of children (AOR= 2.5 95% CI, 1.4, 14.2), and level of education (AOR=3.2 95% CI, 1.1, 9.2) were the factors statistically associated with complete postnatal service uptake. Being healthy was the major (48.8%) reason mentioned for not complying with the recommended three postnatal visits.

**Conclusion:**

The prevalence of complete postnatal care service in the study area was found to be low, and it is far less than the targeted zonal and regional plan. Reinforcing the existing policies and strategies to increase women level of awareness about postnatal care and intensive counseling during antenatal care and delivery are the recommendations based upon the current finding.

## 1. Background

The postnatal period (PNP) is the time beginning immediately following the delivery of the placenta and extending through the six weeks (42 days) of birth. This period represents a critical phase in determining the health and survival of the mother and her newborn [1]. The wellbeing and chance of staying free of morbidity or mortality are much dependent upon the care given during pregnancy, delivery, and most importantly after delivery, the time when many maternal and neonatal deaths take place. Therefore, lack of care during postnatal period may end up in death or morbidity as well as missed opportunities to various healthy behaviors benefiting the mother and her newborn [2, 3]

For both newborns and mothers, the highest risk of death occurs at delivery, followed by the first hours and days after childbirth. It has been shown that more than two-thirds of newborn deaths would occur by the end of the first week after delivery, with up to one-half of all newborn deaths occurring in the first 24 hours. Similarly, approximately two-thirds of all maternal deaths occur in the postnatal period [4, 5].

Although some countries have made a dramatic progress, half of the maternal deaths in the world still take place in Sub-Saharan Africa where little or no progress has been made. Even though there is no single, simple, straightforward intervention that will significantly decrease maternal mortality, several studies have shown that majority of these maternal and neonatal problems could be reduced if women receive appropriate postnatal care [6–9]. In spite of proven benefits of postnatal care on maternal and neonatal health, most newborns and mothers do not receive this service from a skilled healthcare provider during the first few days after delivery; thus, this is the most neglected period for the provision of quality care. Similarly, rates of provision of skilled care are much lower during postnatal period when compared to rates before and during childbirth [3, 10] Likewise, the coverage of postnatal care (PNC) in Ethiopia remains alarmingly limited with only 7% increment over the last one and half decade [11, 12]. Hence, this study aimed at providing a contemporary evidence about the level of PNC coverage and various determinant factors in Northern Shoa, Ethiopia.

## 2. Methods

### 2.1. Study Design and Population

Community based cross-sectional study was conducted from November 2016 to February 2017 with 520 mothers who gave birth within the last ten months preceding the survey. The study was conducted at Debre Berhan town, the capital city of Northern Shoa zone of Amhara regional state located about 130 kilometers to the North East of Addis Ababa (the capital of Ethiopia). Debre Berhan town contains nine urban and five rural kebeles according to administrative classification with estimated population size of 84, 944. More than half 43, 696 (51.4%) of the population in the town are represented by females. Of total females, 26,876 of them are found within reproductive age (15-49) group.

### 2.2. Inclusion and Exclusion Criteria

All women who have given birth within the last ten months preceding the survey were included. Women admitted for much of postnatal periods, those who gave birth greater than ten months ago, and women with still births were excluded from the study.

### 2.3. Sample Size and Sampling Procedure

The sample size for the current study was calculated using single population proportion formula with the assumption of 95% confidence level, 20.2% prevalence of postnatal care utilization [13], 5% marginal error, and design effect of 2. The final sample size (520) was obtained after 5% adjustment for nonresponse rate. The sample size was distributed proportionally for each selected kebeles (the smallest administrative unit in Ethiopia). Finally, systematic random sampling with k^th^ value of five was used to obtain the study subjects in each selected kebele.

### 2.4. Variables and Measurements

The outcome variable was complete postnatal care service utilization which refers to postnatal care service uptake within 24 hours of delivery, the first 3-7 days after delivery, and 7-14th days subsequently. Women who have received the service during all three periods were considered to have complete utilization. The independent variables comprised various sociodemographic (age, educational status, income, and marital status), obstetrics (parity, mode of delivery, ANC use, course of pregnancy, and plan for the current pregnancy), and other health service related characteristics (autonomy, distance from health facility).

The outcome variable was measured by asking the practice and timing of postnatal care visit with three possible responses (i.e., (1) only received postnatal care once after delivery, (2) received twice within the recommended postnatal schedule (successive or interrupted), and (3) received thrice subsequent postnatal care within the recommended time frame). This self-reported visit was cross-confirmed by reviewing postnatal registration records. Finally, the outcome variable was dichotomized as 1: complete PNC utilization and 0: sporadic PNC utilization.

### 2.5. Data Collection and Quality Control

The data were collected using a structured questionnaire via a face-to-face interview at the participant's home. The questionnaire was first prepared in English and then translated into local language (Amharic) and back to English to ensure consistency. The pretest was done on 5% of the total sample size and a necessary adjustment was made. Six midwives and two nurses who are fluent in speaking the local language were involved in the data collection. Two-day training was given to the data collectors and supervisors.

### 2.6. Operational Definition

*Complete Postnatal Visit*. It is for mothers who have stayed in the hospital for 24 hours after delivery and returned for checkup between 3-7 days and 7–14 days subsequently.

*Seclusion*. It is a cultural/social practice that forbids mothers from going out and/or joining peoples for 40 days starting from the day of delivery.

### 2.7. Data Analysis

The data were described using frequency and/or cross-tabulation for categorical variables and mean (standard deviation (SD)) for continuous variables. Binary and multiple logistic regression tests were carried out to identify associated factors at p value threshold < 0.05. The data were coded, cleaned, and entered using EPI-INFO version 7 and analyzed with SPSS version 20. The result was reported strictly following Strengthening the Reporting of Observational Studies in Epidemiology (STROBE) Statement (Supplementary File (available here)).

## 3. Results

### 3.1. Sociodemographic Characteristics

A total of 510 mothers participated in the study making a response rate of 98%. Nearly one-third (31.8%) of respondents were found within the age group of 25–29. Majority of the participants were from Amhara (79.4%) ethic group and orthodox (72.5%) religious background. One-quarter (26.9%) of the mothers attended at least secondary level education and a significant number of women (21.4%) had no any graded education but can read and write (Table 1).

### 3.2. Obstetrics Characteristics

More than half (56.7%) of participants were low multiparous (with children between two and four), whereas nearly third of (29.2%) mothers were primiparous. Some (11.6%) of the mothers reported that the most recent pregnancy was complicated and most (39%) of this problem was attributed to premature rupture of membrane. Cesarean section accounted for 18% of these deliveries and 3.9% of babies born were twins. More than half (53.7%) of participants received the recommended four or more antenatal care service (ANC) (Table 2).

### 3.3. Postnatal Care Service

Only 28.4% of participants received the recommended three postnatal care visits within six weeks of delivery. Among mothers who have visited antenatal care clinic, the majority (58.4%) of these women did not receive counseling about postnatal care. Similarly, only 18.4% of these women initially had information about postnatal care service from different sources. About two-thirds (76.3%) of mothers suggested that there was a social and cultural norm called “Seclusion” which forbids them from coming out of home after delivery. Most importantly almost half (49.1%) of these women respond that this event was more important and valuable than any outdoor visit (Table 3).

### 3.4. Factors Associated with Complete Postnatal Care Utilization

In multivariate logistic regression model, mothers who have given birth through cesarean section were 5.7 [AOR=5.7 95% CI = 3.9 - 19] times more likely to receive complete postnatal care service than those who delivered through spontaneous vaginal delivery. Similarly, mothers having one child (primiparous) were 4.5 more likely to have full postnatal care [AOR= 2.5 95% CI 1.4 – 14.2] than multiparous women. Level of education [AOR=3.2 95% CI, 1.1, 9.2] was another factor which is statistically significant with complete postnatal care utilization (Table 4).

## 4. Discussion

The current study revealed that only 145 (28.4%) of mothers have received complete postnatal care as per the standard recommendation. This finding was slightly higher compared to the reports from other studies [13, 14]. This discrepancy may be attributed to the time gap difference as there would be an improvement on access to healthcare and awareness about the service through time. However, a study conducted in Addis Ababa city administrative by Senait Berhanu and Berhanu wordofa indicated that the postnatal care prevalence was twice of this report (65.6%) [15]. This disparity might be explained by the sociodemographic variation between the study participants such as educational level and living standard as well as nature of the study area including better access to healthcare.

The current study has shown that mothers who attended higher education were three times more likely to receive complete postnatal care service than illiterate women [AOR=3.2 95% CI, 1.1, 9.2]. This finding was in agreement with results from Dembecha district, North west Ethiopia, Nepal, Nigeria, and one more study in Ethiopia [16–19]. This could be explained by the notion that education is a key factor in empowering maternal decision making towards healthcare service, increasing awareness of basic health services, and being informed about health risks, with all of these eventually leading to the improved health seeking behavior.

Cesarean section delivery resulted in increased odds of having complete postnatal service. This finding has also been supported by different studies from Ethiopia and Tanzania [20–22]. This could be because mothers who had operative delivery are tending to have greater perceived susceptibility to a wide range of postoperative complications; therefore, frequent return to the health institution would be the strategy to minimize these perceived risks. Primiparity was another obstetrics determinant for full postnatal care service attendance. Studies in Ethiopia and Nepal have shown consistent finding [19, 20, 23]. This might be justified by the fact that first time mothers are usually dependent upon the support of health professionals and their family on infant care and feeding practice. Similarly, they would be very curious about the health of their newborn baby; thus, these needs might be satisfied through frequent contact with health professionals.

## 5. Limitation of the Study

The result of this study must be interpreted cautiously considering the following inevitable limitations. Though the study has included mothers who gave birth in the last ten months, there might be a possibility of some recall bias. Causality cannot be inferred due to the cross-sectional nature of the study. Moreover, the external validity of the finding might be limited. However, it is the first study which tried to assess the level of complete postnatal care visit in the study area.

## 6. Conclusion

The coverage of full postnatal care service in the study was found to be lower than the targeted zonal and regional plan. Maternal educational status, parity, and mode of delivery were factors significantly associated with postnatal acre service utilization. Reinforcing the existing policies and strategies to increase mother's level of awareness about postnatal care, intensive counseling during antenatal and delivery, and scheduling mothers based on the national postnatal care follow-up protocol to increase postnatal care service utilization were recommended.

## Figures and Tables

**Table 1:** Sociodemographic characteristics of women who gave birth within the last ten months preceding the survey, Northern Shoa, Ethiopia 2017.

**Sociodemographic characteristics **	**Frequency (n)**	**Percent (**%**)**
**Age **		
15 – 19	44	8.6
20 -24	150	29.4
25-29	162	31.8
30 -34	94	18.4
35 and above	60	11.8
**Marital Status**		
Unmarried	30	5.9
Married	456	89.4
Divorced	24	4.7
**Ethnicity**		
Amhara	405	79.4
Tigree	37	7.3
Oromo	39	7.6
Gurage	26	5.1
Others^a^	3	.6
**Religion**		
Orthodox	370	72.5
Muslim	86	16.9
Protestant	41	8.0
Catholic	9	1.8
Others^b^	4	.8
**Educational Status**		
Illiterate	26	5.1
Read and write	109	21.4
Primary Education	125	24.5
Secondary Education	137	26.9
Higher Education	113	22.2
**Husband Education **		
Illiterate	54	10.6
Read and write	72	14.1
Primary Education	111	21.7
Secondary Education	98	19.3
Higher Education	175	34.3
**Household Income **		
< $25	248	48.6
$26 -$99	173	33.9
$100 or more	89	17.5
**Mother occupation **		
House Wife	252	49.4
Self-Employee	100	19.6
Government Employee	114	22.4
Maid servant	32	6.3
Student	12	2.4

^a^Afar and Wolayita; ^b^Bhai and apostolic.

**Table 2:** Distribution of obstetrics history of women who gave birth within the last ten months preceding the survey, in Northern Shoa, Ethiopia 2017.

**Obstetrics characteristics **	**Frequency (n)**	**Percent (**%**)**
**Number of children alive**		
One	149	29.2
Two – four	290	56.7
Five and above	71	13.9
**ANC in recent pregnancy**	454	89
Yes	56	11
No		
**Number of visits **		
One	38	8.4
Two	54	11.9
Three	88	19.4
Four and above	274	60.4
**Course of Pregnancy **		
Complicated	59	11.6
Uncomplicated	451	88.4
**Type of conditions faced**		
Hypertensive disorders	10	16.9
PROM	23	39.0
Post Term	14	23.7
Anemia	7	11.9
Bleeding during pregnancy	5	8.5
**Mode of delivery**		
SVD	356	69.8
SVD assisted with Instrument	62	12.2
Cesarean section	92	18.0
**Number of babies born**		
Single	490	96.1
Twins	20	3.9
**Autonomy on Heath care seeking**		
It's up to me to decide	424	83.1
It's not only me	86	16.9

*∗*ANC: antenatal care; *∗*SVD: spontaneous vaginal delivery; *∗* PROM: premature rupture of membranes.

**Table 3:** Coverage of postnatal care services and related information's among women who gave birth in the last ten months preceding this survey, Northern Shoa, Ethiopia, 2017.

**Variables**	**Frequency (n)**	**Percent (**%**)**
**Postnatal care utilization **		
Complete PNC (3 or more visits)	145	28.4
Sporadic PNC (2 or less PNC)	365	71.6
**Reason for not complying with PNC recommendation**		
I didn't know about its importance	124	33.9
It's a waste of time once we get birth	39	10.7
I waited long time to get the service	57	15.6
Baby and I were completely healthy	178	48.8
Social reasons	84	23
**Counseling about PNC**		
Yes	189	41.6
No	265	58.4
**Previous Information about PNC**		
Had information	94	18.4
Never had information	416	81.6
**Source of Information **		
Media	19	20.2
Magazines or other reading materials	30	31.9
Health extension workers	57	60.6
Friends	13	13.8
**Postdelivery seclusion **		
Yes	389	76.3
No	121	23.7
**Postnatal care vs seclusion **		
It's more important than any clinic visit	191	49.1
No difference to me	103	26.5
No, the visit would be more important	95	24.4

*∗*PNC: postnatal care.

**Table 4:** Bivariate and multivariate analysis of factors associated with complete postnatal care service utilization, Northern Shoa, Ethiopia, 2017.

**Variables**		**PNC**		**Crude OR (95**%**, CI)**	**Adjusted OR (95**%**, CI)**
		Complete	Sporadic		
**Husband education**	Illiterate	15	39	1	1
	Read and Write	15	57	.684 (.300,1.559)	.431 (.151, 1.226)
	Primary education	16	95	.438 (.196,.972	.254 (.091, .706)
	Secondary education	26	72	.939 (.446, 1.98)	.807 (.316, 2.061)
	Higher education	73	102	1.861 (1.25, 3.62)*∗*	1.253 (.531, 2.956)

**Maternal Education**	Illiterate	2	24	1	1
	Read and write	27	82	3.9 (.876, 17.83)	3.1 (.597, 16.9)
	Primary Education	30	95	3.7 (.846, 16.9)	2.5 (.476,13.1)
	Secondary Education	35	102	4.1 (.925, 18.32)	2.02 (.387,10.5)
	Higher education	51	62	6.8 (2.226, 14.34)	3.2 (1.19, 9.2 )*∗*

**Parity**	Primiparous	58	91	2.08 (1.2, 7.8)	2.5 (1.42,14.2)*∗*
	Multiparous	88	202	1.7 (.692, 4.37)	1.76 (.575, 5.39)
	Grande multiparous	24	47	1	1

**Information about PNC**	Had information	32	62	1.34 (1.08,2.23)*∗*	1.98(.553,3.74)
	Had no information	113	303	1	1

**Autonomy on health care**	Self-decision	117	307	1.79 (1.479, 6.30)*∗*	1.27 (.362, 2.261)
	Collaborative decision	28	58	1	1

**Mode of delivery**	SVD	88	268	1	1
	SVD with instrumentation	19	43	3.27 (.927, 4.3)	4.27 (.82,6.2)
	Cesarean section	38	54	2.35 (1.45, 3.81)*∗*	5.7 (3.96, 18.3)*∗*

**Number of babies born **	Singleton	140	350	1	1
	Twins	5	15	.833 (.297, 2.336)	1.025 (.295, 3.558)

**Counseling on PNC**	Received	48	141	.709 (.467, 1.075)	.737 (.446, 1.217)
	Not received	86	179	1	1

*∗*Statistically significant variable; 1 reference category; COR: crude odds ratio; AOR: adjusted odds ratio.

## Data Availability

The data used to support the findings of this study are available from the corresponding author upon request.

## Supplementary Materials

Supplementary Materials
**Supplementary File**. Strengthen the report of observational studies in Epidemiology (STROBE) checklist.
